# Enablers and barriers to implementing cholera interventions in Nigeria: a community-based system dynamics approach

**DOI:** 10.1093/heapol/czae067

**Published:** 2024-07-26

**Authors:** Kelly Elimian, Karin Diaconu, John Ansah, Carina King, Ozius Dewa, Sebastian Yennan, Benjamin Gandi, Birger Carl Forsberg, Chikwe Ihekweazu, Tobias Alfvén

**Affiliations:** Department of Global Public Health, Karolinska Institutet, Stockholm, Sweden; Exhale Health Foundation, Abuja, Nigeria; Institute for Global Health and Development, Queen Margaret University, Edinburgh, United Kingdom; Center for Community Health Integration, School of Medicine, Case Western Reserve University, Cleveland, United States; Department of Global Public Health, Karolinska Institutet, Stockholm, Sweden; School of Health Systems and Public Health, University of Pretoria, Pretoria, South Africa; Nigeria Centre for Disease Control and Prevention, Abuja, Nigeria; Bauchi State Ministry of Health, Bauchi State, Nigeria; Department of Global Public Health, Karolinska Institutet, Stockholm, Sweden; Nigeria Centre for Disease Control and Prevention, Abuja, Nigeria; Department of Global Public Health, Karolinska Institutet, Stockholm, Sweden; Sachs’ Children and Youth Hospital, South General Hospital, Stockholm, Sweden

**Keywords:** Cholera interventions, community, healthcare system, Nigeria, political will

## Abstract

Nigeria accounts for a substantial cholera burden globally, particularly in its northeast region, where insurgency is persistent and widespread. We used participatory group model building workshops to explore enablers and barriers to implementing known cholera interventions, including water, sanitation and hygiene, surveillance and laboratory, case management, community engagement, oral cholera vaccine, and leadership and coordination, as well as exploring leverage points for interventions and collaboration. The study engaged key cholera stakeholders in the northeastern States of Adamawa and Bauchi, as well as national stakeholders in Abuja. Adamawa and Bauchi States’ group modes building participants comprised 49 community members and 43 healthcare providers, while the 23 national participants comprised government ministry, department and agency staff, and development partners. Data were analysed thematically and validated via consultation with selected participants. The study identified four overarching themes regarding the enablers and barriers to implementing cholera interventions: (1) political will, (2) health system resources and structures, (3) community trust and culture, and (4) spill-over effect of COVID-19. Specifically, inadequate political will exerts its effect directly (e.g. limited funding for prepositioning essential cholera supplies) or indirectly (e.g. overlapping policies) on implementing cholera interventions. The healthcare system structure (e.g. centralization of cholera management in a State capital) and limited surveillance tools weaken the capacity to implement cholera interventions. Community trust emerges as integral to strengthening the healthcare system’s resilience in mitigating the impacts of cholera outbreaks. Lastly, the spill-over effects of COVID-19 helped promote interventions similar to cholera (e.g. water, sanitation and hygiene) and directly enhanced political will. In conclusion, the study offers insights into the complex barriers and enablers to implementing cholera interventions in Nigeria’s cholera-endemic settings. Strong political commitment, strengthening the healthcare system, building community trust and an effective public health system can enhance the implementation of cholera interventions in Nigeria.

Key messages

- Political will emerges as a complex influence on implementation and must be understood in the broader health system and socio-political and development context.
- Health system resources for cholera intervention implementation are broadly acknowledged as limited, and existing centralized structures exert further substantive pressures on access and co-ordination of care.
- Trust in the health system is compromized: this directly affects community uptake of cholera interventions and willingness to access care.
- Traditional leaders are favourably positioned to advocate for improved political will, and religious leaders are favourably positioned to facilitate the implementation of cholera interventions in the community.
- COVID-19 directly resulted in the implementation of WASH interventions like those needed for cholera and demonstrated the potential for action.

## Introduction

Globally, there are at least 2.9 million cholera cases and 95 000 cholera-related deaths every year (Ali *et al*., 2015), with low- and middle-income countries accounting for most of the figures due to suboptimal access to potable water, sanitation and hygiene (WASH) (World Health Organization, 2019). Between 4 January and 14 November 2021, seven West African countries, including Nigeria, Benin Republic, Burkina Faso, Cameroon, Mali, Niger and Togo, reported 108 859 cholera cases and 3711 deaths (case fatality ratio: 3.4%) (Sodjinou *et al*., 2022). Notably, Nigeria was the most affected country, accounting for 95% of the cholera cases (Sodjinou *et al*., 2022). In contrast to earlier outbreaks in Nigeria with a narrow geographical spread (Dalhat *et al*., 2014; Elimian *et al*., 2019), 33 of 37 Nigerian States (including the Federal Capital Territory) reported cholera cases and 3298 deaths (with a case fatality ratio of 3.5%) from October 2020 to October 2021, underscoring the country’s increased vulnerability to cholera outbreaks (Elimian *et al*., 2022). Similar to previous cholera outbreaks in Nigeria (Dalhat *et al*., 2014; Elimian *et al*., 2019), the country’s North-East region was the most affected during this recent cholera outbreak, calling for increased focus on understanding the enablers and barriers to implementing cholera interventions in the region.

The Global Task Force on Cholera Control (GTFCC) launched and adopted the ‘Ending Cholera: A Global Roadmap to 2030’ strategy in 2017, aiming to eliminate cholera in at least half of the current endemic countries and reduce cholera-related deaths by 90% by 2030 (Global Task Force on Cholera Control, 2017). Attaining these goals hinges on implementing six multi-stranded cholera interventions, including WASH, surveillance and laboratory, oral cholera vaccine (OCV), healthcare system/case management, community engagement, and leadership and coordination. Nigeria’s most recent cholera outbreak suggests the country is not yet on track to actualizing the goals outlined in the global roadmap strategy (Sodjinou *et al*., 2022). Furthermore, elimination requires countries to assess their capacities across these domains, including identifying constraints, challenges and bottlenecks to implementation (Global Task Force on Cholera Control, 2020). Our assessment of healthcare facility capacity to implement these interventions in Adamawa and Bauchi States, North-East Nigeria, found resource availability to be low and varied within States and by cholera interventions, highlighting the need for a context- and intervention-specific approach to strengthen Nigeria’s capacity for cholera control (Elimian *et al*., 2023). However, the research provided us with the relevant baseline data to inform stakeholder engagement in order to strengthen the implementation of the cholera interventions, a necessity for effective and sustainable implementation in the region.

A participatory approach involving the engagement of crucial cholera stakeholders in identifying the enablers and barriers to implementing the cholera interventions is crucial (Elimian *et al*., 2023). However, there is no evidence of stakeholder engagement to understand enablers and barriers to implementing multi-stranded cholera interventions in Nigeria or outside Nigeria. Therefore, this study engages cholera stakeholders at various levels (community, State and national) to fill this gap, while identifying leverage points for strengthening Nigeria’s capacity to respond to recurrent cholera outbreaks. Findings from this study will be directly helpful to local, national and global cholera policymakers and actors in planning the development and implementation of locally-appropriate cholera control interventions for Nigeria.

## Methods

### Study design and theoretical framework

We conducted a series of Group Model Building (GMB) workshops with key cholera actors underpinned by community-based participatory frameworks (Tremblay *et al*., 2018). GMB is an established systems thinking methodology for engaging stakeholders to gain a mutual understanding of complex problems, thus enhancing buy-in from stakeholders and, ultimately, the chances of accepting and implementing recommendations from the developed model. GMB works with stakeholders to deeply and actively involve them in model construction through the exchange, assimilation and integration of mental models into a holistic system description (Urwannachotima *et al*., 2019). When used under the auspices of system dynamics or other similar methods, GMB models can also help in understanding the non-linear behaviour of complex systems over time, recognizing the value of engaging relevant stakeholders directly in order to generate findings that are contextually relevant and implementable (Andersen *et al*., 2007; Gerritsen *et al*., 2020).

### Overview of multi-stranded cholera interventions

The multi-stranded cholera interventions are outlined in Table 1.

**Table 1.** Description of multi-stranded cholera interventions

Cholera intervention	Description
Water, sanitation, and hygiene (WASH)	WASH is considered the long-term solution for cholera control and hinges on ensuring safe water, basic sanitation and good hygiene practices.
Surveillance and laboratory	Cholera surveillance is hinged on adequate epidemiological data and laboratory diagnostics (e.g. rapid diagnostic test and/or culture) capacity at various data collection and feedback levels.
Case management	Case management encompasses the treatment of cholera patients through prompt administration of oral rehydration solution (ORS) and, for severe illness, as characterized by dehydration, rapid administration of intravenous fluids and appropriate antibiotics in a manner that is safe for both patient and healthcare workers.
Oral cholera vaccine (OCV)	Supported by Gavi (the Vaccine Alliance), OCVs are typically made available for reactive mass vaccination campaigns in areas with endemic cholera, in humanitarian crises with a high risk of cholera and during cholera outbreaks, preferably in conjunction with other cholera interventions such as WASH and community engagement.
Community engagement	Community engagement refers to factoring in the people, communities and local culture into developing and implementing cholera interventions.
Leadership and co-ordination	Multi-sectoral leadership and co-ordination is responsible for harnessing and mobilizing resources to effectively identify needs and implement cholera interventions to meet the identified needs.

### Study setting

**Figure 1.**
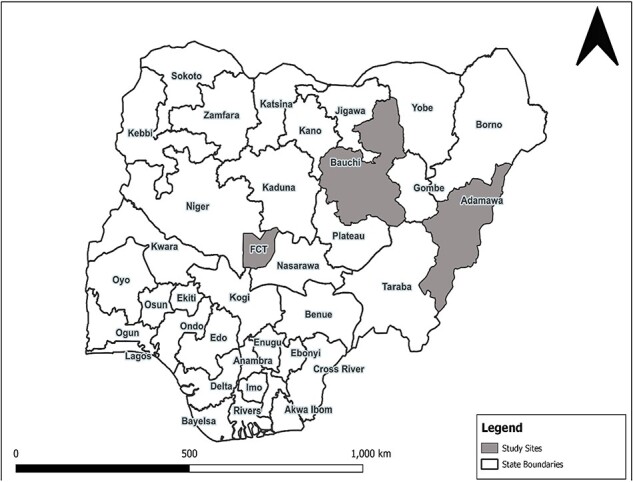
Map of Nigeria showing the study locations (grey)

Nigeria comprises 36 States and the Federal Capital Territory (Abuja), with each State further disaggregated into several Local Government Areas (LGAs). This study was conducted in Adamawa and Bauchi States in the North-East region (Figure 1). Additionally, the study was conducted in Abuja to capture the perspectives of national cholera stakeholders. Adamawa and Bauchi States were selected because of their high cholera endemicity (Elimian *et al*., 2019), having consistently accounted for a substantial number of cholera cases and deaths during the recent cholera outbreaks in Nigeria. Bauchi recorded 19 453 cholera cases during the 2021 cholera outbreak in Nigeria, the highest figure across the country (Elimian *et al*., 2022); both Adamawa (2748 cases and 41 deaths) and Bauchi (9405 cases and 35 deaths) States were among the top five most affected States during the 2018 cholera outbreak in Nigeria (Elimian *et al*., 2019). Additionally, we selected these States for the study to capture the varying degrees of fragility, which impacts public health by limiting a population’s capacity to respond and adapt to stressors and shocks (Diaconu *et al*., 2020), such as a cholera outbreak. Some LGAs in Adamawa State had been directly attacked by Boko Haram insurgents, while some LGAs in Bauchi State serve as host communities to persons displaced from Boko Haram-affected neighbouring States, including Adamawa, Borno and Yobe.

### Sampling

We adopted a purposive sampling approach to recruit participants with the purpose of capturing diverse genders, ages (≥18 years), occupational groups, experiences related to cholera, and persons with active engagement in cholera response and control across the study locations.

### Data collection

First, we conducted key informant interviews with relevant cholera stakeholders at various levels of governance (community, State and federal/national) to understand the enablers and barriers to implementing cholera interventions from their perspectives. The lead researcher conducted the interviews using semi-structured interview guides customized for each study participant group (Table 2), previously piloted with healthcare workers and community members in Abuja for clarity. The interviews were recorded, transcribed and analysed using a thematic approach (Braun and Clarke, 2006). The preliminary findings from these interviews were used to inform the GMB workshops with each participant group.

**Table 2.** Overview of data collection methods

Location	Description of data collection method	Semi-structured interviews	GMB workshops
**Adamawa**	Number of participants	24	43 22 Community members (15 men and 7 women) 21 Healthcare providers (16 men and 5 women)
	Number of Workshops/dates	N/A	2 Community stakeholders: 24 August 2021 Health providers: 26 August 2021
	Example of participants	Community members: previous cholera patients and caregivers, farmers, local food retailers/market women, school teachers, local health promoters, and community and religious leaders Health providers: disease notification and surveillance officers, health educators, community health extension workers, nurses, clinicians, and staff of government ministries, departments and agencies (MDA; e.g. State ministries of health, water resources, environment, and primary health care development agency), cholera technical and development partners (e.g. WHO, Medecins Sans Frontieres)	Community members: community leaders, religious leaders (Imam and Pastor), students, market women representatives, public transporter, school teacher, youth representative, and ex-cholera patients Health provider: community extension health workers, disease notification and surveillance officers, State Ministry of Health and WHO surveillance staff, nurses, laboratorians, WASH representative from the State Ministry of Water Resources and United Nations International Children's Emergency Fund, clinicians, and NGO representatives
**Bauchi**	Number of participants	17	49 27 Community members (21 men and 6 women) 22 Healthcare providers (19 men and 3 women)
	Number of workshops/date	N/A	2 Community members: 17 August 2021 Health providers: 19 August 2021
	Example of participants	Same as those in Adamawa	Same as those in Adamawa
**Abuja**	Number of participants	7	23 (10 females and 13 males)
	Number of workshops/date	N/A	1 National stakeholders: 12 August 2021
	Example of participants	National choler stakeholders: government MDA staff (staff of the Nigeria Centre for Disease Control and Prevention, federal ministries of water resources, environment, and primary health care development agency, cholera technical and development partners (WHO, International Federation of Red Cross and Red Crescent Societies, United Nations International Children's Emergency Fund, US Centre for Disease Control and Prevention)	National stakeholders: 12 August 2021: Staff of Nigeria Centre for Disease Control and Prevention, Federal Ministries of Water Resources and Environment, United Nations International Children's Emergency Fund, International Federation of Red Cross and Red Crescent Societies, US Centre for Disease Control and Prevention, WHO, cholera researcher. Additionally, staff of the GTFCC Cholera Support Platform were present as observers

N/A: Not applicable

The second data collection phase adopted a system dynamics approach and used GMB workshops (Hovmand, 2014). The GMB scripts were adapted from Scriptapedia (Scriptapedia, 2020), drawing on the preliminary findings from the key informant interviews. We conducted five all-day GMB workshops, one each with community participants in Adamawa and Bauchi States and one each with healthcare providers in the same States; the last workshop was with national cholera stakeholders in Abuja. We chose participant-specific GMB workshops to minimize potential power dynamics and to recognize differences in views or beliefs about cholera and its drivers by different groups of participants. GMB participants were similar to those interviewed in the key informant interviews but not entirely the same individuals were interviewed (Table 2).

Each GMB workshop included an introduction of the research team and participants by the Convener (e.g. Director of Public Health); an overview of the study and an introduction to the use of GMB methods by the lead researcher; and specific GMB activities for the day. The lead researcher and trained research assistants were assigned specific or dual roles according to standard GMB protocol (Hovmand, 2014), as outlined in supplementary File 1 (see online supplementary material). The scripts for each GMB workshop across locations are presented in supplementary File 2 (see online supplementary material). The GMB workshops utilized three interactive exercises and tools: (1) trends over time, (2) cognitive mapping and (3) a causal loop diagram.

#### Trends over time

**Figure 2.**
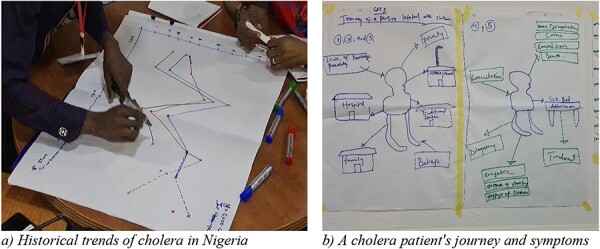
Participants drawing the historical trends of cholera. (a) Historical trends of cholera in Nigeria. (b) A cholera patient's journey and symptoms

We asked participants to share their understanding of cholera epidemiology and their perceived risk factors in the community or country over the past 10 years. Using an empty graph on a flip-chart with time on the *x*-axis and measures of disease burden (e.g. incidence, deaths etc.) on the *y*-axis, the research team guided the participants to draw their perceptions of how the aforementioned variables have changed over time (Figure 2). The participants were prompted with questions such as: ‘What is the trend of cholera in your community since the 2015 presidential election in Nigeria?’, ‘What is the typical journey of a cholera patient within your community?’, ‘What are the risk factors influencing the transmission of cholera in your community?’, ‘What interventions are available for cholera control in your community and/or Nigeria?’ In addition to drawing the historical trends of cholera, participants were asked to identify two future pathways they predicted would occur if current cholera trends continued or if interventions occurred.

#### Cognitive mapping

This visual tool sought to introduce participants to systems thinking by exploring their understanding of the enablers/barriers to implementing cholera interventions and the consequences of implementing these interventions successfully or not (Gerritsen *et al*., 2020). A cognitive mapping template was developed and provided for the participants to complete this activity.

#### Causal loop diagram

The causal loop diagram captured the dynamic interrelationships of an issue and the presence of feedback in systems (Gerritsen *et al*., 2020). Here, feedback loops, a primary operating unit of systems (Meadows, 2008), were captured by connecting the variables identified by participants’ previous activities (e.g. barriers to implementing cholera interventions and consequences of action and inaction) guided by the cause-and-effect relationships. To ensure that the interrelationships identified in causal loop diagrams represent participants’ consensus, participants were asked to reflect on their previous responses outlined in the combined causal loop diagram. These involved participants adding, deleting and modifying the relationships (structures) in the map. Finally, we explored potential interventions to address the barriers to implementing cholera interventions. For this, we asked the following questions to probe for possible actions to address the identified barriers: ‘What variables could you increase or decrease?’, ‘How could you impact connections: strengthen or weaken a connection, speed it up or slow it down, add or delete connections?’ This required the participants to write potential actions on post-it notes and place them on the variable in the causal loop diagram where they might be introduced to achieve the desired outcome in the diagram. Finally, all the participants were required to select the top three actions for each cholera intervention in which their group would like to see progress.

### Data analysis

As described by participants in their initial concept models, connections between variables were translated into an electronic model using the VenSim PLE x64 software (Ventana Systems, 2022). GMB models underwent iterative analyses. Still using preliminary findings from the key informant interviews and notes from the GMB sessions, variables and pathways in the concept models were refined and, as needed, consolidated to ensure that the concept models reflected the causal logic of participants. In any case of unclarity in the developed models, we contacted 2–3 representatives of the GMB workshop for verification. The resulting causal loop diagrams underwent further iterative critical analyses. The research team compared models developed across the different workshops and groups and further consolidated information from these models into individual overarching causal loop diagrams, separately for community members, healthcare providers, and national cholera stakeholders. This process involved comparing variables and their definitions and pathways to ensure consistent and divergent information was captured. Finally, reinforcing and balancing loops (a reinforcing loop amplifies change to the system while a balancing loop counteracts change) were identified in the causal loop model where appropriate. Further, we discussed the leverage points for intervention with the GMB participants and considered the potential actions identified as points for interventions aimed at strengthening the implementation of cholera interventions.

## Results

During analysis of the final casual loops, it became evident that findings across the healthcare provider, community and national stakeholder workshops focused on four main domains directly related to aspects that influence how cholera interventions are coordinated and implemented. Additionally, findings from community members and health providers did not differ by State, hence the chosen mode of results presentation. A summary of the key findings relative to each theme is provided in Table 3. Cholera interventions in the casual loop diagrams are in boxes.

**Table 3.** Summary of key study findings

Theme	Main findings
Domain 1: perceptions around political will	Lack of political will results in (1) limited financing and allocation of resources needed for the implementation of cholera interventions and (2) limited leadership and coordination by government authority as relates to cholera interventions. Reasons for lack of political will include (1) lack of incentive around coordination and cooperation and (2) lack of interest in diseases related to broader—and difficult to address—determinants of health (poverty). The problem is perpetuated by lack of M&E frameworks and their implementation, which would enable accountability towards populations.
Health system resources and structures	Directly affected by the above lack of political will and financing, there is a lack of resources needed (technology, internet connectivity) to detect, manage and report on cholera. This results in lack of interest by local structures and stakeholders to maintain response platforms for outbreaks; but in emergency cases, multiple actors then mobilize, but often duplicate efforts. While guidelines for cholera management exist and knowledge is high, enablers for detection are lacking at the primary healthcare level. At the secondary care level, centralized management of cholera cases was recognized as being of mixed benefit.
Community trust and culture	Community trust and ownership are critical in assuring receptiveness to messages in emergencies and in overcoming vaccine hesitancy. Unsustainable interventions, duplication of effort and lack of coordination negatively affect trust in the formal health system. Community cultural beliefs and practices can inhibit uptake of certain cholera interventions.
Spill-over effect of the COVID-19 pandemic	The whole-of-government approach implemented has a significant positive spillover on cholera, both by generally ensuring further investments in health and also by enhancing specific areas. For example, the strengthening of surveillance systems for COVID-19 also enhanced cholera surveillance, which is a precursor for ordering OCV. The COVID-19 response demonstrates that political will can be mobilized.

### Domain 1: perceptions around political will

Healthcare providers and community members perceived all three tiers (local, State and federal) of governments’ main responsibility as ensuring the adequacy and quality of intervention. Both groups expressed predominantly negative views of the government regarding these responsibilities, noting that political will relating to cholera intervention coordination and implementation was lacking. The main reasons healthcare providers cited for this were miscommunication by government officials, misappropriation of resources and insufficient focus on curbing cholera transmission (e.g. urban planning and enforcement of relevant sanitation practices). Box 1 offers an example of healthcare providers’ views on WASH interventions specifically.

Box 1: Healthcare providers’ views on political will relating to WASH

**Figure 3.**
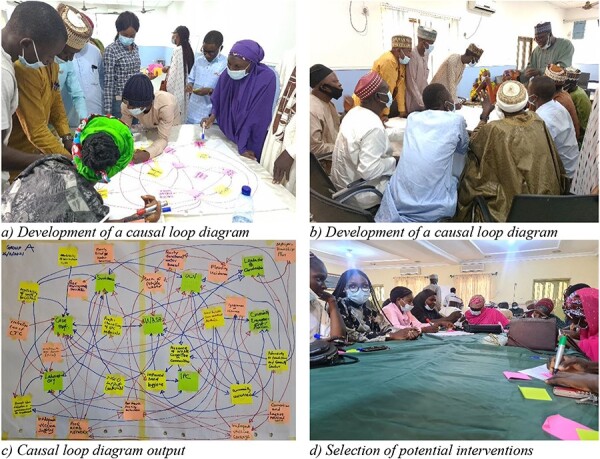
Causal loop diagram development process and outputs. (a, b) Development of a causal loop diagram. (c) Causal loop diagram output. (d) Selection of potential interventions. Pictures were taken with the participants’ written consent to publication

Healthcare providers unanimously agreed that inadequate political will by government leadership in the three-tiers of governance (local, State and federal) is the most critical determinant of the outcome of WASH service implementation in the study locations. They also believed that the importance of government political will for improved WASH service is in ensuring both adequacy and quality. Participants specified that inadequate political will for WASH services has about four implications (Figure 3, upper corner right).

- Promotes poor communication among Ministries: some participants cited an example of when the Ministry of Environment failed to inform partners, including the Ministry of Water Resources, about the urgent need to replace broken drainage pipes observed during an inspection of a community water supply in Bauchi State.
- Promotes the misappropriation of WASH resources, including funding. This is a scenario referred to as ‘corruption’ by some participants. Participants believed that inadequate political will subtly promotes ‘corruption’ due to weak leadership in promoting transparency and accountability for resources in the WASH sector.
- Contributes to the non-implementation of urban planning and policy. Participants noted that poor urban planning increases the risk of cholera transmission during the rainy season when drainage and road networks become easily blocked by refuse and cause the contamination of open water sources (e.g. open-wells, rivers and streams).
- Weakens the enforcement of environmental sanitation practices favourable for cholera prevention and control. Participants cited the abandonment of the ‘environmental sanitation’ (cleaning houses and the immediate environment every Saturday morning with restrictions on movement) that used to be commonplace in Nigeria.

Lack of political will was noted to be balanced by active involvement (in terms of funding and technical support) of multi-lateral organizations, such as the United Nations International Children's Emergency Fund UNICEF) and the World Health Organization (WHO).

Community members echoed healthcare providers, explaining that the health sector severely lacked resources in the form of laboratory capacity (thus directly impacting cholera surveillance and control), and trained healthcare workers and relevant equipment (thus compromising case management). Community members also discussed how inadequate political will manifests as limited monitoring and evaluation (M&E) guidelines for cholera intervention implementation.

**Figure 4.**
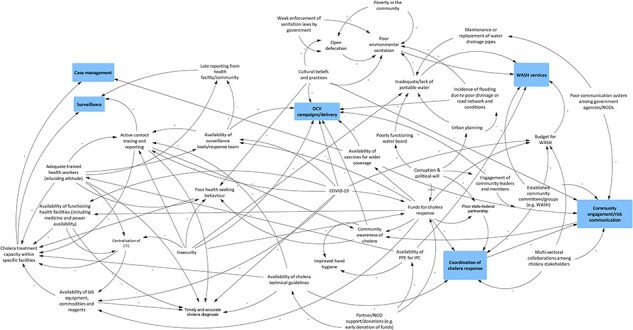
Output from community GMB workshops

**Figure 5.**
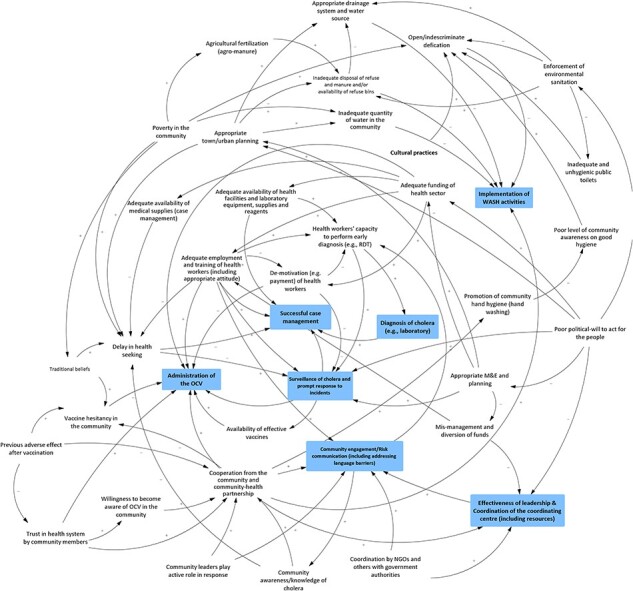
Output from healthcare providers GMB workshops

Further, both healthcare providers and community members believed that inadequate political will directly affects the leadership and co-ordination of cholera technical groups or emergency operation centres during an outbreak. Technical and financial support from donors and multinational non-governmental organizations (NGOs) (development partners) at the bottom of Figure 4 significantly influences cholera outbreak response by governments at various levels. According to healthcare providers, this is the reason for State and federal governments’ complacency to invest in cholera outbreak preparedness and response. Healthcare providers noted that, in collaboration with government MDAs at both federal and State levels, development partners often take the lead in funding and developing technical or standard operating guidelines (SOPs) for cholera surveillance, case management, risk communication, and infection prevention and control activities. To further highlight the high degree of government complacency, some healthcare professionals cited instances when State government MDAs failed to facilitate the distribution of already developed guidelines by development partners to public health personnel at the local government level. Although community members shared similar views to those of healthcare providers (Figure 5, lower right), they noted that inadequate political will sometimes discouraged development partners from providing support, such as pipe-borne water, to communities, which culminates in community distrust in the healthcare sector.

National stakeholders also perceived inadequate political will as a challenge to cholera intervention coordination and implementation. Alongside poor knowledge of the disease and its burden by federal government policymakers and politicians, national stakeholders’ explanations situate the need for more political will in a macro-level policy context.

First, national stakeholders noted that inadequate political will must allows for the proliferation of parallel programmes and/or overlapping policies by federal government MDAs and development partners. Participants noted that MDAs and partners often justify this situation as protection of mandates or interests. Consequently, coordinating cholera interventions, such as WASH, could be challenging and sometimes counterproductive. Participants noted that cholera-reporting States are often negatively affected by inter-agency rivalry at the federal level of governance as they are forced to choose an agency or partner to work with.

Second, in alignment with community and health-provider perceptions, national stakeholders agreed that political will was a determinant of funding for implementing cholera interventions. According to national stakeholders, political will is more important than the funding itself, given that its availability depends mainly on political will. They supported this belief by citing the all-of-government response to the coronavirus disease (COVID-19) pandemic in Nigeria, which attracted interest from the three tiers of government and consequently channelled substantial resources into indigenous public health response.

Third, inadequate political will was believed to promote a lack of ownership of cholera response by State governments and their relevant MDAs, often characterized by bureaucratic hurdles in releasing and accepting resources for cholera response (sometimes the activity may be abandoned).

Fourth, again, in agreement with the perceptions of the communities consulted, national participants believed that inadequate political will allows for the non-integration or absence of the M&E framework in implementing cholera interventions. They noted that a lack of M&E hinders accountability for scant cholera resources, allows for the implementation of substandard interventions (e.g. healthcare workers’ training), and contributes to non-adherence to established guidelines or SOPs by healthcare workers.

### Domain 2: perceptions around healthcare system resources and structures

Healthcare providers discussed several factors believed to impact the implementation of cholera interventions directly, classified as corresponding to healthcare systems resources (e.g. availability of medical technology, tools, trained workforce) or structures (i.e. how the health system organizes cholera services).

One of the first issues providers and community members discussed is the availability of appropriate equipment at health facilities. Providers emphasized that the scarcity of laboratory commodities is a recurring challenge to cholera confirmatory diagnosis (culture) at the State level. This situation was partly attributed to the epidemic or unpredictable nature of cholera outbreaks, discouraging suppliers from stockpiling commodities in order to minimize financial loss. Participants cited several instances of cholera-specific laboratory commodities expiring in health facilities and suppliers’ stores.

Community members commented on limited medical equipment and supplies (e.g. oral rehydration solution and rapid diagnostic test kits) in healthcare facilities, which affect the health facility’s capacity to deliver robust cholera case management. This was noted to discourage healthcare-seeking by community members with suspected cholera, given the perceived limited capacity of healthcare facilities (especially those operating at the primary level) to undertake cholera diagnosis (upper left-centre of Figure 5).

Healthcare providers spoke about facilities lacking the necessary internet connectivity to facilitate cholera surveillance according to the Integrated Disease Surveillance and Response system. Specifically, participants noted that intermittent internet connectivity or, in most cases, lack of internet, affects the timeliness of reporting cholera surveillance data within and outside the State. This challenge was reported to be frequent despite the availability of tools (e.g. tablets with digital data collection software installed) for surveillance activities. Participants also identified tools that support cholera surveillance in their locations, such as the availability of context-specific guidelines with varying definitions of cholera using local languages, which promotes the standardization of cholera surveillance. Healthcare providers noted that the tools are particularly crucial given the variation in surveillance capacity within States and across the country. For instance, cholera surveillance was believed to be more efficient in urban than rural areas.

Healthcare providers also discussed how healthcare workers’ attitudes and training influence the implementation and success of cholera intervention. Participants emphasized that healthcare workers’ poor attitude, both to work and patients, contributes to delays experienced by cholera patients, who could be perceptive to subtle hostility from healthcare workers—this was also believed to negatively affect healthcare facility-based cholera surveillance as many cholera cases could be missed due to non-presentation for formal capturing by the surveillance system. However, participants acknowledged that certain factors beyond the control of healthcare workers, such as insecurity, community beliefs and practices, and community level of cholera awareness, play a significant role in determining the timeliness of healthcare seeking.

Participants noted two main challenges relating to health system structures. The first is the centralization of cholera care, whereby cholera treatment centres within urban State-owned specialist hospitals or federal medical centres could be detrimental or beneficial to cholera case management. On the one hand, it can potentially enhance the clinical outcome of cholera patients, given the ease of coordination and concentration of resources for cholera case management, usually in a single cholera treatment centre. On the other hand, it could worsen the clinical outcome of cholera patients due to self-medication and delays in travelling a long distance from peripheral areas (usually rural or peri-urban areas) to such treatment centres in urban areas. According to community members, the frequent referral of patients to a higher level of care (government secondary or tertiary hospitals) or private hospitals (profit-oriented) contributes to community members’ distrust of the healthcare system.

A second issue related to current structures and processes within the health system is poor coordination and communication for cholera response. Healthcare providers noted that self-interest among government MDAs at the federal and State levels and development partners has a deleterious impact on cholera response coordination, especially for the emergency operation centre during a cholera outbreak (Figure 4, lower right). Participants buttressed this by noting that the multi-sectoral coordination platform for cholera is usually inactive before or after a cholera outbreak due to members’ reluctance to attend meetings and the need for more funding to facilitate meetings for preparedness and planning. Consequently, planning for prepositioning essential commodities and community engagement activities before a cholera outbreak is usually suboptimal, and there is an increased tendency for inter-agency rivalry.

Poor communication between government MDAs and development partners at both federal and State levels was viewed as a natural extension of the above-noted problem, posing a challenge to community engagement, including cholera risk communication messaging (Figure 4, lower right). Participants believed that poor communication between MDAs is driven mainly by interest in access to and managing financial resources. Participants noted that the situation often results in the duplication of efforts or programmes, further wasting the scarce resources for cholera control.

**Figure 6.**
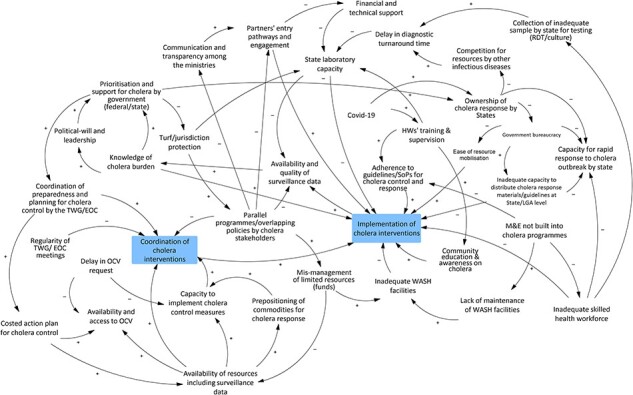
Output from national stakeholders GMB workshop

National stakeholders also believed that the existing bureaucratic process in requesting OCVs from GAVI through the GTFCC delays OCV campaigns, resulting in inadequate vaccines to meet community needs (Figure 6). Participants, however, noted that the OCV request process could be enhanced by improving the quality of in-country surveillance data, which is a core requirement for a costed action plan.

### Domain 3: perceptions around community trust and culture

All participants acknowledged that community trust in the formal healthcare system is crucial for implementing cholera interventions. Participants noted that engaging community leaders and members actively, irrespective of socioeconomic status or affiliation, and respectfully is essential to ensuring implementation.

Community members were convinced that community trust in federal and State government MDAs and development partners is crucial to community engagement in cholera control activities (Figure 4, bottom centre). They noted that an engaged community is more receptive to health interventions (e.g. participation in contact tracing) and willing to seek healthcare, thus enhancing cholera response and clinical outcomes.

In highlighting the critical role of trust and community ownership, community members, for example, cited instances where WASH interventions championed by State government and international NGOs failed to be sustained shortly after exiting the community due to a lack of community ownership of the intervention, or in some settings, community refusal to use the WASH services provided (e.g. chlorinated water or public toilet). The latter scenario often occurs when rumours about the poor safety of available WASH services are not counteracted by adequate information from community members.

Healthcare providers further noted that community cultural beliefs and practices play an essential role in determining the outcome of an intervention. When discussing targeting services, such as campaigns against open defaecation in the study locations (Figure 3, upper right), providers noted that cultural beliefs in some communities promoted open defaecation even when public water toilet cisterns were available. The other detrimental belief providers identified is the misconception that vaccines are a means of controlling population growth.

Healthcare workers noted that while beliefs may, on the one hand, be harmful, communities have huge capacities, which, if mobilized, could help the implementation of cholera interventions. For example, healthcare workers believed that insecurity, often mediated by bandits and/or Boko Haram insurgents, threatens the implementation of OCV campaigns in the study locations. However, some participants mentioned how community members have contributed to improving access to healthcare amidst increasing insecurity. They do this by voluntarily providing real-time security information to healthcare personnel and security agencies and occasionally forming a local vigilante group to complement the existing security system. Furthermore, community participants (Figure 3, lower left) believed that the delivery of OCV campaigns would be much easier if measures, such as the engagement of traditional and religious leaders and contextualization of risk communication to local settings (e.g. using local dialects), were promoted in order to convince community members about the benefits of the intervention. For community leadership, they believed efforts should also be made to maximize the relationships between religious leaders (closer to the people) and traditional leaders (closer to the politicians) to promote the implementation of cholera interventions.

### Domain 4: perceptions around the spill-over effect of the COVID-19 pandemic

The Nigerian ‘all-of-government’ approach to the COVID-19 pandemic response was acknowledged by many participants to have had significant effects on cholera and its response. For example, healthcare providers believed that the COVID-19 pandemic had unintended benefits for WASH services delivery in healthcare facilities and communities in the study locations. Moreover, participants believed that community COVID-19 committees, established to promote WASH services (e.g. through locally-led campaigns), played a crucial role in improving community WASH services for cholera.

Healthcare providers also believed that investment in COVID-19 surveillance (e.g. training and retraining of healthcare workers and provision of tools and funding for internet subscription) unintentionally improved cholera surveillance. The existence of the Integrated Disease Surveillance and Response system in the country mediated this process. National stakeholders also supported this view.

In turn, improved cholera surveillance—partly due to investments in COVID-19 response—was believed to enhance the ease of OCV requests from GAVI through the GTFCC (robust surveillance data is part of the requirements for OCV requests). However, healthcare providers noted that increasing COVID-19 vaccine hesitancy in Nigeria might extend to the vaccination of other diseases, including oral cholera vaccine, an issue believed to be more prominent when healthcare workers’ capacity for risk communication of vaccine type, route of administration, potential benefits and side effects is suboptimal.

National stakeholders, in particular, believed the substantial financial investments in the public health sector during the pandemic could motivate States to take ownership of the cholera response, albeit the investment for cholera response might need to be commensurate with that for COVID-19. Notably, they believed that the experience of the COVID-19 pandemic could change the historical narrative of States’ reluctance to disclose cholera cases for fear of socioeconomic repercussions (Figure 6).

## Discussion

We identified a range of enablers and barriers to implementing cholera interventions from the perspectives of stakeholders with diverse roles, locations and experiences (community members, healthcare providers, national actors and development partners) in Nigeria’s fragile and cholera-endemic States. The inclusive and participatory nature of the GMB approach facilitated participatory discussions on this critical disease that are of relevance to global health security. Our findings were grouped into four overarching domains, including perceptions around (1) political will, (2) healthcare system resources and structures, (3) community trust and culture, and (4) the spill-over effect of the COVID-19 pandemic.

### Political will: a bridge to cholera-control action

This study identified how inadequate political will affects the implementation of cholera interventions by weakening accountability and monitoring of compliance with SOPs, promoting inter-agency rivalry, and reducing financial commitment and accountability by government cholera stakeholders, thereby diminishing cholera outbreak preparedness and response coordination systems. As reported in this study and supported by a study on cholera outbreak response in Haiti in 2010 (Ferreira, 2020), reduced government funding for cholera management and response results in a ‘hollow state’—a State that relies on development partners for joint or independent public service delivery with little coordination (Goldsmith and Eggers, 2004). Therefore, our study has given the concept of political will more analytical precision by highlighting how public health actors can use an understanding of the impact of political will on the delivery of public health services to co-create and enhance the implementation of cholera interventions more equitably.

Enabling the availability of more resources for cholera control interventions was identified as a change lever in cholera control, the inverse of which was found to have detrimental effects, like findings of the GTFCC in which limited resources for cholera control activities were identified as a barrier hampering the implementation of cholera endemic countries’ National Cholera Action Plans (Global Task Force on Cholera Control, 2020). With enhanced political will and the resultant resource increase, communities can be empowered to effectively implement cholera interventions. Further, our findings indicate that recurrent cholera outbreaks, with attendant high morbidity and mortality in Nigeria, are more of a political than a development issue, particularly concerning WASH, surveillance and laboratory, case management, and coordination interventions. Community members and healthcare providers supported this notion by citing political commitment, which is also evidenced in a study that explained how the ‘all-of-government’ approach aided the COVID-19 response in Nigeria (Abubakar *et al*., 2021).

Community leaders, such as Emirs or Kings, are considered opinion leaders, given their influential roles in political organizations (Gaitho, 2019) and active engagements during cholera outbreaks in Nigeria (News Agency of Nigeria, 2021) and elsewhere (Bracquemont and Abdulkhaliq, 2019). Thus, they are favourably positioned to improve political will for cholera control in Nigeria. However, community members in the present study believed that religious leaders, such as Imams or Priests, may be even more influential than community leaders in enhancing political will, given the high value placed on religion in Nigeria. Therefore, it is pertinent to leverage the indigenous and influential roles of both religious and traditional leaders in negotiating for better political will for the implementation of cholera interventions, especially at the community level, in cholera endemic areas in Nigeria.

### Health system challenges as an existential threat to implementing cholera interventions

Suboptimal capacity for cholera surveillance and laboratory diagnosis in the study States were noted to derail cholera surveillance activities, a finding similar to observations in Nepal (Rhee *et al*., 2020), as well as delay cholera outbreak declaration and response, as evidenced in Borno State in 2017 (Ngwa *et al*., 2020). The existing protocol for requesting OCV from GAVI was considered by government cholera stakeholders at the national level to be bureaucratic, given the many application hurdles, and subject to the limitations of the cholera surveillance system within a country. While the bureaucratic challenge is not surprising as it corroborates the current shortage of OCV stockpiles globally (Pezzoli and Oral Cholera Vaccine Working Group of the Global Task Force on Cholera, 2020), the application outcome’s dependence on surveillance data could negatively impact community trust in public health system initiatives like OCV campaigns, contact tracing and WASH (M’Bangombe *et al*., 2018).

Inter-agency rivalry and its attendant impact on coordination mechanisms is a well-known issue in the Nigerian security sector (Odoma, 2014); however, our findings indicate that it is a significant barrier to implementing cholera interventions at both federal and State levels. Although the issue seems more prominent at the federal level (experiential) than the State level (inferential), participants noted that the high level of autonomy of both federal and State government MDAs and health facilities explains this trend. As noted by participants at the State level, lack of clarity on healthcare facilities’ terms of reference for coordinating cholera outbreak response also promotes the reported rivalry. However, beyond our findings, Ngwa and colleagues, during the 2017 cholera outbreak in Borno State of Nigeria, noted that rivalry among cholera case management actors stemmed from concerns regarding recognition and credit for work (Ngwa *et al*., 2020). Lastly, the centralization of cholera care within specialist or tertiary hospitals in the State capital, albeit promoting a coordination mechanism, was considered a healthcare system structure that implicitly promotes rivalry and eventual hoarding of resources, including crucial surveillance data for decision-making and planning.

Beyond the formal healthcare system, community members underlined their innate capacity to perceive and easily detect hostility from healthcare workers. Such observations encourage self-medication or deter prompt healthcare-seeking, especially if suspected cholera is deemed non-severe. This attitudinal issue from healthcare workers has also been noted in the context of HIV in Africa (Magak, 2022). Therefore, the negative consequences of healthcare workers’ attitudes, including the indirect promotion of antimicrobial resistance (Jani *et al*., 2021) and home-management of cholera, should be highlighted in training and retraining interventions.

### The moderating role of community trust and culture in cholera control

During a disaster, community trust in the local government can influence the public’s perceptions of risks associated with the crisis and enhance their controllability (Woskie and Fallah, 2019). Thus, investing in strategies to understand community perceptions and awareness is crucial for building community trust for the successful implementation of community-based cholera interventions (Lemay-Hébert, 2014). However, our findings suggest that while community perceptiveness and awareness are indeed crucial in this regard; they are distinct. The former is more innate and a major determinant of community trust, while the latter is acquired and is largely a product of educational attainment. Understanding these subtle differences between community perceptiveness and awareness of cholera is even more critical for the North-East region of Nigeria, where a substantial number of forcibly displaced persons may be less trusting of formal institutions (Woskie and Fallah, 2019).

Community culture was also identified as a strong determinant of community trust, essential in the study locations with cultural, linguistic and geographical heterogeneities. Therefore, cholera stakeholders need to promote a robust sociocultural discourse around cholera in the contexts of community culture, as expressed in ideas, beliefs, fears and help-seeking behaviour. Such an approach is crucial in implementing effective, acceptable and sustainable cholera interventions (Schaetti *et al*., 2009; Said *et al*., 2011). This will help address common misconceptions around OCVs, such as their being the cause of infertility, as reported in some Tanzanian communities (Schaetti *et al*., 2009), and potentially promote the healthcare system’s resilience thanks to improved partnerships between healthcare facilities and community stakeholders (Meyer *et al*., 2020).

### Spill-over effects of the COVID-19 pandemic

Many of our study participants, including community members, mentioned that the impact of the COVID-19 pandemic response in Nigeria on implementing cholera interventions was two-pronged. On one hand, the pandemic put an additional strain on the healthcare system and its capacity to promptly and effectively respond to a cholera outbreak, particularly in the study locations where the healthcare system is known to be weaker than that in other regions in the country (Çavdaroğlu *et al*., 2022). Few participants noted that the federal and State governments neglected cholera and other infectious diseases of public health importance at the pandemic’s peak. On the other hand, healthcare providers believed that implementing cholera interventions, particularly WASH services, benefited from investments in COVID-19 prevention and control measures. Additionally, the resources provided to States’ governments in response to the pandemic were believed to have enhanced cholera surveillance, with an attendant positive impact on subsequent estimation of cholera cases during this period. Historically, cholera cases tend to be underestimated by States or governments owing to travel restrictions and isolations and implications for trade and tourism (Ganesan *et al*., 2020).

#### Recommended strategies to enhance the implementation of cholera interventions

Most national cholera stakeholders believed establishing a National Cholera Control Programme would be an effective and sustainable approach to holistically address most of the identified challenges to implementing cholera interventions in Nigeria, particularly the North-East. This was observed during the COVID-19 pandemic when most governments effectively established national coordination mechanisms (World Bank, 2022). According to the stakeholders, a National Cholera Control Programme backed by the three-tiers of governments (federal, State and local) with technical and financial support from development partners (e.g. the GTFCC-led Cholera Support Platform) can enhance the co-ordination of cholera outbreak preparedness and response as well as address the counterproductive inter-agency rivalry. Nigeria is making plans in this direction by proposing a preliminary framework for a National Cholera Steering Committee, chaired by the country’s Vice-President and co-chaired by the Ministers of Health and Water Resources, while the heads of relevant MDAs and the Governors Forum are engaged. This approach, with the necessary political support, to a large extent mirrors Zambia’s top-bottom approach (WHO Regional Office for Africa, 2019), where coordination of the national cholera control programme is domiciled in the Vice-President’s Office (WHO Regional Office for Africa, 2019); however, findings in the present study underline the need to adopt a multisectoral approach (e.g. One Health) and engage State government and community representatives so that the proposed initiative does not become over-centralized at the national level, with little or no impact at the State and community levels where the actual response to cholera outbreaks occur. In addition, given the significant influence of flooding owing to poor town planning, the Ministry of Land, Housing and Urban Development at the federal and State levels needs to be engaged as a critical partner, alongside the Ministries of Health, Environment and Water for cholera control in Nigeria. Other recommendations by the study participants based on perceived ‘impacts’ and ‘ease of implementation’ at various study levels are outlined in supplementary File 3, see online supplementary material).

#### Strengths and limitations

To our knowledge, this is the first study to provide a contextually grounded description of enablers and barriers to implementing cholera multi-stranded interventions from the perspectives of stakeholders with diverse roles, locations and experiences in cholera-endemic settings. Therefore, the findings add significant value to the renewed commitment to the global roadmap goals. Moreover, findings from this study are formative and help generate new hypotheses, and the causal loop diagram offers a lens to explore potential leverage points that may take the form of a policy, programme or intervention that strengthens Nigeria’s strategies for cholera control. Nonetheless, this study has limitations. The healthcare providers in our GMB workshop in Bauchi State might be biased towards men; we attempted to mitigate this by creating a separate group for the female participants. However, the sampled population might reflect the actual distribution of healthcare providers in the State due to the predominance of the Islamic religion in the State. Additionally, the qualitative nature of the GMB approach is considered a limitation by experts in systems dynamics, hence the advocacy that researchers quantify postulated causal relationships established in the qualitative models using quantitative models (Mui *et al*., 2019). Therefore, we will seek to adopt a quantitative simulation model using historical cholera surveillance data to further the evidence from the present study.

## Conclusion

This study offers insights into the diverse and complex barriers and enablers to implementing cholera interventions in cholera-endemic settings in Nigeria. Strong political commitment, adequate health system resources and structures, community trust in the federal and State government and an effective public health system all influence the implementation of cholera interventions. Additionally, the COVID-19 pandemic had positive and negative effects on cholera interventions but has critically illustrated the potential for mobilizing resources and political will to address a health priority.

## Supplementary Material

czae067_Supp

## Data Availability

The data underlying this article will be shared upon reasonable request by the corresponding author.

## Reflexivity statement

We made a conscious effort to promote the principle of inclusivity and balance in terms of gender, seniority and regional location throughout the conduct of this research.

The authors comprise eight researchers who identify as men (KE, JA, OD, SY, BG, BCF, CI and TA) and two who identify as women (KD and CK). In addition, in recruiting participants for the study, especially the group model building workshops, we adopted gender-specific grouping such that female participants were in a separate group from their male counterparts. This was to minimize the potential influence of a dominating voice, especially in northern Nigeria where Islam is the predominant religion, on the findings.

In terms of seniority, the authors come from diverse professional backgrounds, ranging from early-mid career researchers (KE and OD) to established researchers (KD, JA, CK, BCF and TA), as well as public health professionals, including State Epidemiologist (BG), Public Health Director (SY) and Assistant Director General (CI).

Regarding regional location and affiliations, the authors come from Nigeria, including public health institutions in specific locations where the study was conducted (SY, BG and CI), Sweden (KE, CK, BCF and TA), South Africa (OD), and the UK (KD) and USA (JA). Additionally, the lead researcher (KE) is a Nigerian, though based in Sweden. All authors have extensive experience with systems, qualitative research and health policy, and systems dynamics in low- and middle-income countries.

Lastly, we were sensitive to potential bias from the research team, hence the acknowledgement that our perspectives may influence the causal loop diagrams and findings. This is because researchers from diverse backgrounds collaborated on synthesising the causal loop diagram and brought various positions and perspectives, including practical experiences from several cholera outbreaks, besides those of the participants.
