# Supplementary material for: Enablers and barriers to implementing cholera interventions in Nigeria: a community-based system dynamics approach
Source: Health Policy Plan. 2024 Jul 26;39(9):970–84. doi: 10.1093/heapol/czae067 (PMC11474597; doi:10.1093/heapol/czae067)
Supplement: czae067_Supp [file czae067_supp.zip › SUPPLEMENTARY FILE 1.docx]

**Supplementary file 1**

| **Description of roles for GMB workshops** | |
| --- | --- |
| Convener | To open the workshop– preferably someone who has status among the participants- and help set the tone for the workshop |
| Group facilitators | To facilitate the activities and to organize the outcomes of each activity into thematic clusters; to transfer products from participants to modellers. |
| Modeler/reflector | To develop the qualitative model and then reflect on model-based insights with the participants |
| Recorders/ translators | To take notes and document activities from each GMB session, including drawings of models and dynamics of the model; to provide simultaneous translation between local language and English during activities as needed. |
| Closer/Debriefer | To give concluding remarks summarising the findings of the workshop; to lead and facilitate the debrief of the facilitation team after the workshop |
| Choreographer | To conceptualize and oversee the overall design of the GMB workshop, including developing the detailed agenda, scripts, and pre-workshop training. |
| Observers | To observe but not participate in the process. |
