# Supplementary material for: Enablers and barriers to implementing cholera interventions in Nigeria: a community-based system dynamics approach
Source: Health Policy Plan. 2024 Jul 26;39(9):970–84. doi: 10.1093/heapol/czae067 (PMC11474597; doi:10.1093/heapol/czae067)
Supplement: czae067_Supp [file czae067_supp.zip › SUPPLEMENTARY FILE 2.docx]

**Supplementary file 2**

## Community stakeholders (Adamawa and Bauchi States)

| **Script 1: GMB with communities** | |
| --- | --- |
| **Time (min)** | **Activity** |
| 5 | **Opening Prayers** |
| 10 | Introduction of the study team and participants by the Convener |
| 20 | **Overview of the study**  The group facilitator will define the term “cholera” and briefly explain the concept of “fragility” and “fragile settings”. He will clearly state that cholera share similar clinical features and risk factors with other diarrhoeagenic diseases, but emphasise the acuteness of cholera (however, he will not mention the risk factors as these variables will be identified by the participants). |
| 60 | **Rich pictures** **and reference modes**  **Split participants into 2 groups and ask each group:**   - To draw what does “cholera” mean? - Why is cholera present in your community? Prompt participants to detail their perceptions of cholera and common risk factors. - Do they believe that there is an interaction with health providers to prevent this?   **In the past 10 years, depict the:**   - Number of cholera cases per year in their community - Number of cholera-related deaths in their community - Number of people with cholera seeking formal health care   Rating (on a scale of 1-10) of the quality of the response of the health system to cholera outbreaks.  Note: No right or wrong answers; use bold lines for actual estimates and dotted lines for projections: look out for 2020 and ask questions as needed  **Draw the journey of a person infected with cholera seeking support:**   - What happens when someone has cholera? - Where do they go to seek help and support? Why do they do this? - If someone seeks help at the health system, what happens here? - How do you know someone is well after having cholera? - What happens to those with severe cases of cholera?   *Ensure to ask: what would be the determinants of this health seeking behaviour? What are the facilitators and barriers to get successful results (i.e. the patient stops acute stooling)?*  *What is easier/more difficult in a rural/urban area? Are there any differences that come to mind?*  *If not mentioned, ask the participants about key cholera risk factors such as access to clean water and hygienic practices.*  *Also, prompt participants to explore the issue of trust in community members (who do people trust the most) and in formal healthcare system.* |
| 45 | **Variable elicitation**  Ask participants to split into the same groups used in the previous activity. Ask each group to use sticky notes and brainstorm:   - What are the factors that negatively affect the implementation of cholera interventions? Why do cholera interventions fail in practice? Are these factors the same for all the interventions? Are there any specific interventions? - What about positive factors? What helps makes the implementations of cholera interventions a success? Also helps to refer to it again from the rich pictures. - If required, prompt participants to identify variables regarding the current knowledge status of cholera interventions by their communities. Where you have community leaders, ask how much of the identified barriers/facilitators are being driven by community members themselves and by health professionals/government? How they contribute towards improving these within their communities? Do they need to add more variables accordingly?     *No specific ‘intervention’ will be discussed, but participants will be prompted to think of activities/influence they already hold and how they have used this previously. The research team will describe initiatives during the present cholera outbreak in the State (e.g. risk communication) to strengthen the response to cholera– community members and leaders will be offered the chance to reflect on such initiatives and offer advice/critique.*  *Variables will be compared among each other to ensure clarity of their meanings and avoid duplication. Participants may be asked to provide definitions for some variables by the modelling team.* |
| 30 | **Break**  *Research team members will put together all the findings of previous activities. They will identify a set of variables to be used in developing the following activities.*  *Minimise the variables generated; choose two first variables and explain how they are connected.* |
| 60 | **Causal loop diagram development (Part 1)**  Participants will be invited to identify how the identified variables from the previous activity **enable** or **block** the implementation of cholera interventions [generally first and then specifically] and how these variables contribute to securing a “healthy community / community safe from cholera” in their community, explicitly illustrating them using the sticky notes [i.e. how do these variables lead to a healthy community (community members may not understand a successful/unsuccessful implementation of cholera interventions)].  The research team will ensure that the causal links, proposed by the groups, are clear and justifiable by them. Peer-review of the models by other group members will be encouraged with a possibility to elaborate one final model, and researchers will be interrogating the causal nature of links each time a new link is proposed. |
| 60 | **Lunch** |
| 60 | **Causal loop diagram development (Part 2)**  Each group model will be projected on the screen or front board. Each part of the model will be presented separately, and members of each group will be invited to comment on the accuracy of the model. Changes will be made accordingly whenever a consensus is guaranteed. |
| 60 | **Identifying leverage points**  Now that you have the model, participants are asked to look at it and reflect:  How would you fix the things that you say are not working? Are there new ways to ensure the health of the community or protect the community against cholera infection?  What actions would you take? Can you put them on post-it notes and map them onto the model (place them in the appropriate place on the flip chart)?  Also apply the principle of impact and ease of implementation. |
| 5 | **Closing prayer** |

## GMB with healthcare professionals, technical partners and donors (Adamawa and Bauchi)

| **Time (min)** | **Activity** |
| --- | --- |
| 5 | National Anthem |
| 10 | Opening remarks and introduction of the study team and participants by the Convener |
| 10 | Study overview and demonstrating use of GMB methods |
| 60 | Rich **pictures**  Split participants into 2 groups and ask each group:   - Draw the journey of a person infected with cholera seeking medical support (where does he get diagnosed? ER, pharmacy, GP, …)? - Fill in the drawing with a pre-infection drawing – what were these patients like/doing before/as they arrive to clinic? - What would be the determinants of this health seeking behaviour (i.e. what are the facilitators and barriers to this health seeking behaviour)? - What are the facilitators and barriers to get successful results or recovery?   *If not mentioned, ask the participants about key cholera risk factors.*   - What happens to patients with severe cholera? - Is this patient journey different during a major cholera outbreak, like now? If yes, how is the journey different? - Who are the players in the management of the patient infected with? In the response and control of a cholera outbreak?   ***Reference modes***  Participants will be asked to draw graphs representing the last 10 years. Graphs will focus on:   - Number of cholera cases - Number of diagnosed cholera cases of the total suspected cases - Number of deaths from cholera infection - Number of cholera patients seeking formal health care - Number of “success stories” - Priority rating (on a scale of 1-10) cholera has received over time in terms if comparison to other diseases and availability of resources for interventions   At the end, ask groups’ representatives to present the rich pictures and graphs to each other.  Participants might be invited to think of potential changes around cholera epidemiology due to insurgency in the North-East. |
| 20 | **Break** |
| 30 | **Variable elicitation**  Ask participants to split into the same groups used in the previous activities. Ask each group to use sticky notes and brainstorm:   - Factors negatively affecting the implementation of cholera interventions in general (15 minutes) Are any specific types of cholera interventions? - Factors positively affecting the implementation of cholera interventions (15 minutes) Are any specific types of cholera interventions?   Encourage them to brainstorm: how could patients’ experiences change according to health workers’ capacity to respond or manage cholera? Do they need to add more variables accordingly?  Variables will be compared among each other to ensure clarity of their meanings and avoid duplication. Participants may be asked to provide definitions for some variables by the research team. Ensure neutral wording of the variables. |
| 15 | **Inter-relationship diagram**  Participants will be invited to identify the links between the variables and explicitly illustrate them using the sticky notes from the previous activity. |
| 60 | **Causal loop diagram development (Part 1)**  In no particular order, ask participants to depict cholera interventions on a flipchart using sticky notes of the same colour. Participants will be invited to identify how the identified variables from the previous activity enable or block the implementation of cholera interventions (generally and/or specifically) and how the variables identified contribute to securing a “healthy community / community safe from cholera” in their state, explicitly illustrating them using the sticky notes.  The research team will ensure that the causal links, proposed by the groups, are clear and justifiable by them. Peer-review of the models by other group members will be encouraged with a possibility to elaborate one final model, and researchers will be interrogating the causal nature of links each time a new link is proposed.  *Bearing in mind the different stages by which the population goes from being healthy to getting cholera, participants will be invited to add the variables to this model focusing on how the health system is currently dealing with these stages of cholera outbreak response.* |
| 30 | **Causal loop diagram development (Part 2)**  One model will be projected on the wall. Each part of the model will be presented separately, and all the group will be invited to comment on the accuracy of the model. Changes will be made based on group discussion, and areas of consensus identified but also divergent views will be captured. |
| 60 | **Lunch** |
| 30 | **Identifying leverage points/fragility points**  The research team will identify along with participants’ points of ‘fragility’ (weak points) for cholera interventions and their likeliness to benefit from interventions. Ask participants: Where would you interfere or like to intervene? What is the easiest to change? Where would we achieve most impact with changes?  Use a different colour of sticky note as needed. |
| 60 | **Identification /prioritization of interventions**  Ask the groups to identify as many ideas/interventions as they can for interventions that could impact the previously identified points of fragility/leverage.  On each group’s tables, participants will be asked to rate these ideas/interventions according to their impacts and ease of implementation in their context. **These ideas/interventions will be placed on a wall with two axes** (y-axis: impact; x-axis: ease of implementation).  High Impact    Ease of implementation  (-) (+)  Low Impact  Discussions around the facilitators and barriers to the proposed ideas/interventions will be prompted as well. |
| 5 | **Closing remarks by the Convener** |

## National stakeholder workshop

This workshop will be focusing on the bigger picture of things which inhibit cholera response in Nigeria and specifically in the North-east. We will be aiming to understand:

- What is the current system behind coordinating/implementing cholera interventions from national level perspective?
- What are barriers and facilitators (to be compared to the findings from other study phase) and what can be changed?
- Are there new broader actions or interventions that can address these change areas?

| **Time (min)** | **Activity** |
| --- | --- |
| 5 | Nigeria National Anthem (Second Stanza) |
| 5 | Goodwill message from the Director General of NCDC |
| 5 | Goodwill message from Global task Force on Cholera Control (GTFCC) |
| 10 | Welcome and introduction of the study team and participants by the Convener |
| 20 | Overview of Country Support Platform (CSP) |
| 10 | Overview of the study, including the use of GMB methods |
| 30 | **Rich pictures**  Working in 2-3 groups (7-10 persons per group), participants draw graphs over time (e.g. in the last 10 years) depicting:   - Incidence/frequency of cholera outbreaks (*bold line*) and expected trajectory of cholera outbreaks (*dash line*) for the future (2030 as per the GTFCC roadmap) - Drivers of recurrent cholera outbreaks in Nigeria (specifically in the North-east?) - **Priority** rating (on a scale of 1-10) cholera has received over time (compared to other conditions, such as COVID-19) and **availability** (on a scale of 1-10) of resources for cholera interventions (WASH, case management, surveillance/laboratory, OCV, coordination and leadership, risk communication/community engagement). For this latter one, 1-2 lines only will be needed or straight lines, with the y-axis length indicating the level of rating, but participants need to agree on the most relevant thing to plot.   Participants then share their graphs over time to each other and reflect on key trends and events that influence how the graphs evolve. |
| 20 | **Tea break** |
| 30 | **Variable elicitation**   - Looking back at the rich pictures, and thinking about the current system behind **1.** **coordinating and 2. implementing cholera interventions** from national level perspective, **each** participant is asked to identify 2-3 **variables/factors** that are relevant to **each** (on post-its). Alternatively, ask: what things affect the success or failure, directly and indirectly, in achieving cholera intervention coordination and implementation? - Individual *vs* collective interventions? - **All** participants are then invited to take turns, **share one variable** and in discussion to refine the phrasing of the variable. Duplicate variables need to be removed. - After all variables are discussed, they are **all placed on a whiteboard/paper** (separated by group) so that participants can work with them in the next exercise. - Facilitator clusters the post-it into themes while the recorder for the session writes down the list of all causal variables and take pictures. |
| 45-60 | **Mapping the current system for coordinating and implementing cholera interventions**   - The modelling team will present participants with preliminary concept model structures (reference modes) as elaborated from preliminary findings from KIIs and health facility surveys.   For example, this concept model can include some key variables that correspond to barriers and facilitators to coordination of cholera interventions or to implementation thereof. Example below:  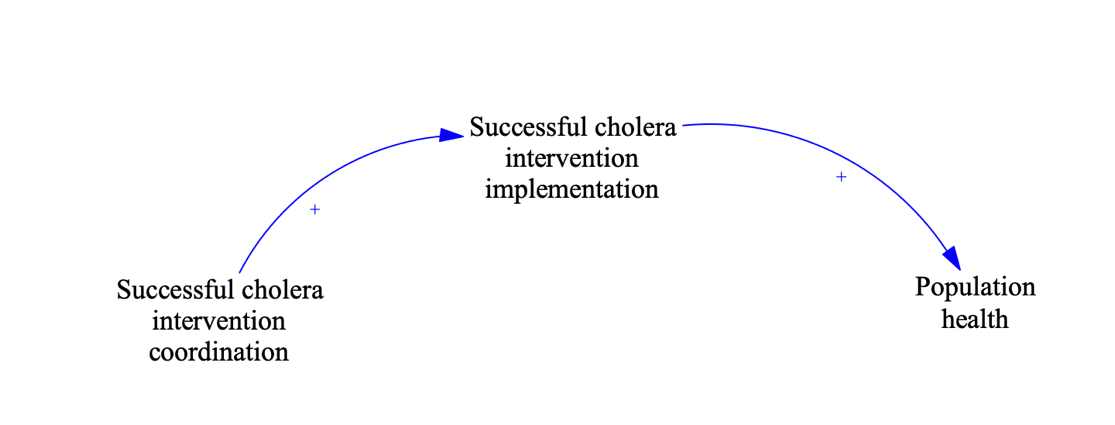   - Participants are then asked to use the variables they came up with (on the board) and connect variables in a causal loop diagram building from the seed model.   Whenever facilitators or barriers are discussed, it is key to **prompt**:   - Do these barriers/facilitators apply to all cholera interventions (e.g. surveillance, WASH, community engagement etc.) or to specific ones? - Are there specific barriers/facilitators that occur in NE Nigeria as opposed to other areas? - At the end of the development of the causal loop diagram, participants will be asked to discuss in plenary which areas of the diagram are most amenable to intervention: which barriers could be addressed or which new actions could be put in place to secure better outcomes regarding cholera. |
| 30 | **Lunch break** |
| 45-60 | **Policy and intervention mapping**   - Using the developed models and areas identified for action from the previous session, the modelling team will ask participants to draw on/elaborate any key policies/interventions currently implemented and desired (Participants should distinguish current policies/interventions from desired policies/interventions using different coloured post-it sheets) in relation to the identified areas from previous session. Again, participants will be encouraged to work in groups during this task. - The modelling team will prompt participants to carefully consider the implications of any interventions, including positive and negative spill-over effects (I.e. thinking about what the interventions being proposed may promote more widely or may hinder; e.g. focusing more on better planning of land-use and making water safe, may mean that some funds are moved from WASH to this), coordination mechanisms and implications on resource use. - Participants will be asked to rate these policies/interventions according to their impact and ease of implementation in (1) Nigeria and (2) North-east Nigeria. These interventions/policies will be placed on a wall with two axes (y-axis: impact; x-axis: ease of implementation). - The modelling team will provide participants with small cards (in different colours) to facilitate the listing of interventions.   High Impact    Ease of implementation  (-) (+)  Low Impact |
| 10 | **Closing remarks by the Convener and closing prayers** |
