# Supplementary material for: Enablers and barriers to implementing cholera interventions in Nigeria: a community-based system dynamics approach
Source: Health Policy Plan. 2024 Jul 26;39(9):970–84. doi: 10.1093/heapol/czae067 (PMC11474597; doi:10.1093/heapol/czae067)
Supplement: czae067_Supp [file czae067_supp.zip › SUPPLEMENTARY FILE 3.docx]

**Supplementary file 3**

| **Strategies to enhance the implementation of cholera multi-stranded interventions in Nigeria** | |
| --- | --- |
| National stakeholders | *Co-ordination* |
|  | Establish a high-level (top-bottom) co-ordination platform for the control of cholera and other diarrhoeal diseases |
|  | Identify in real-time and prioritise cholera hotspot areas in distributing WASH resources and OCV |
|  | Strengthen the public health emergency operating centre (PHEOC) network |
|  | Improve the co-ordination and mapping of resources for cholera control |
|  | *Implementation* |
|  | Strengthen community ownership of cholera interventions |
|  | Enhance surveillance and improve data quality |
|  | Enhance the provision of WASH services in health facility and communities |
|  | Conduct laboratory capacity assessment and strengthening |
|  | Improve risk communication based on context-specific research evidence |
| Healthcare providers | Training and retraining of healthcare workers (including addressing poor attitudes to work/patients and providing them with essential tools/updated guidelines) |
|  | Actively engaging community members in planning and implementing cholera interventions. |
|  | Improved collaboration and communication between partners, especially in the area of prepositioning of diagnostic reagents/commodities. |
|  | Re-introduction of environmental sanitation to the community |
| Community members | Engage religious leaders to improve cholera risk communication and sensitisation (including the engagement of locals for interpretation of messages) |
|  | Organise and enforce regular community sanitation activities, including designated points for refuse collection and disposal |
|  | Deploy trained and equipped healthcare workers for cholera response |
|  | Good governance and increased political-will regarding cholera control |
|  | Organise community group/committee for accountability and quick response to a cholera outbreak |
|  | Inclusion of women in the community leadership structure |
